# Supplementary material for: Nudging policymakers on gendered impacts of policy
Source: PLoS One. 2023 Oct 19;18(10):e0293036. doi: 10.1371/journal.pone.0293036 (PMC10586654; doi:10.1371/journal.pone.0293036)
Supplement: S1 File — (DOCX) [file pone.0293036.s001.docx]

**Supporting Information**

**S1 File. Briefing email invitations in the control and treatment conditions, and briefing slides.**

(Control condition) As you know, gender equity isn’t just a “women’s issue”. Everyone in your community and the NWT benefits if policy and legislation doesn’t unfairly impact one gender more than others.

Click “accept this invitation” and join this **10-minute briefing on gender and policymaking in the NWT** to find out what it means in your work and to the people of the NWT. At the end of the briefing, you will also have a chance to make a pledge of your commitment to gender equity.

To attend this briefing, click on the following link.

Join Zoom Meeting: [link]

If you would like to attend but cannot make this date or time – *please select your preferred time from this list* and a new invitation will be sent: [link]

Marsı | Masi |Kinanāskomitin | Thank you | Merci | Hąį’ |Miigwetch| Quana , Qujannamiik | Quyanainni | Mahsı| Mahsı̀,

Janet Dean(she/her)

Policy, Projects and Outreach Specialist

Status of Women Council of the NWT, 4th Floor, Northwest Tower

PO Box 1320 | 5201 Franklin Avenue | Yellowknife, NT X1A 2L9

The Status of Women Council of the NWT has joined forces with a team from the University of British Columbia’s Behavioural Insights program to run a study exploring how policymakers and influencers consider important issues like gender equity. By participating in this study, you’ll play an active and visible role in improving the lives of all genders in the NWT. We are asking you to make a public declaration of your commitment to gender equity as part of this study.

If you have any questions, please reply to this email. **You have been personally selected to receive this invitation. We ask that you please don’t share it with others at this time.**

(Treatment condition) As you know, gender equity isn’t just a “women’s issue”. Everyone in your community and the NWT benefits if policy and legislation doesn’t unfairly impact one gender more than others.

Click “accept this invitation” and join this **10-minute briefing on gender and policymaking in the NWT** to find out what it means in your work and to the people of the NWT. At the end of the briefing, you will also have a chance to make a pledge of your commitment to gender equity.

**Gender equity in policy means real things to real people.**

Agnes has recently left her abusive partner and would like to have her own home. The Home Purchase program in her community requires a good credit history, but Agnes and many women like her don’t have a good enough credit history because their partner kept the finances in their name and/or accumulated a bad credit history in her name. This means the housing program unfairly impacts women like Agnes and many others who have experienced economic abuse. Housing policies could be revised to consider this unique situation for Agnes and other women like her.

**Why you make a difference**

Emily is a carpenter who had to leave her previous job due to discrimination and sexual harassment. Emily dreamt of opening her own construction business so she could create a different work culture for herself and others. Gaining her Blue Seal Certification would help her do this. However, she has 2 kids at home and can’t afford to leave the NWT to gain the necessary certification. When policymakers in the NWT decided to expand the Minister’s authority to issue Blue Seal Certifications within the NWT, it meant Emily and those like her now have greater access to opportunities like these and the NWT has more local businesses. By making this policy “gender-blind”, all genders benefit.

To attend this briefing, click on the following link.

Join Zoom Meeting: [link]

If you would like to attend but cannot make this date or time – *please select your preferred time from this list* and a new invitation will be sent: [link]

Marsı | Masi |Kinanāskomitin | Thank you | Merci | Hąį’ |Miigwetch| Quana , Qujannamiik | Quyanainni | Mahsı| Mahsı̀,

Janet Dean(she/her)

Policy, Projects and Outreach Specialist

Status of Women Council of the NWT, 4th Floor, Northwest Tower

PO Box 1320 | 5201 Franklin Avenue | Yellowknife, NT X1A 2L9

The Status of Women Council of the NWT has joined forces with a team from the University of British Columbia’s Behavioural Insights program to run a study exploring how policymakers and influencers consider important issues like gender equity. By participating in this study, you’ll play an active and visible role in improving the lives of all genders in the NWT. We are asking you to make a public declaration of your commitment to gender equity as part of this study.

If you have any questions, please reply to this email. **You have been personally selected to receive this invitation. We ask that you please don't share it with others at this time.**

(Briefing slides) The slides were identical in both conditions, except that slide 4 contained the personal stories and was only present in the treatment condition.

**
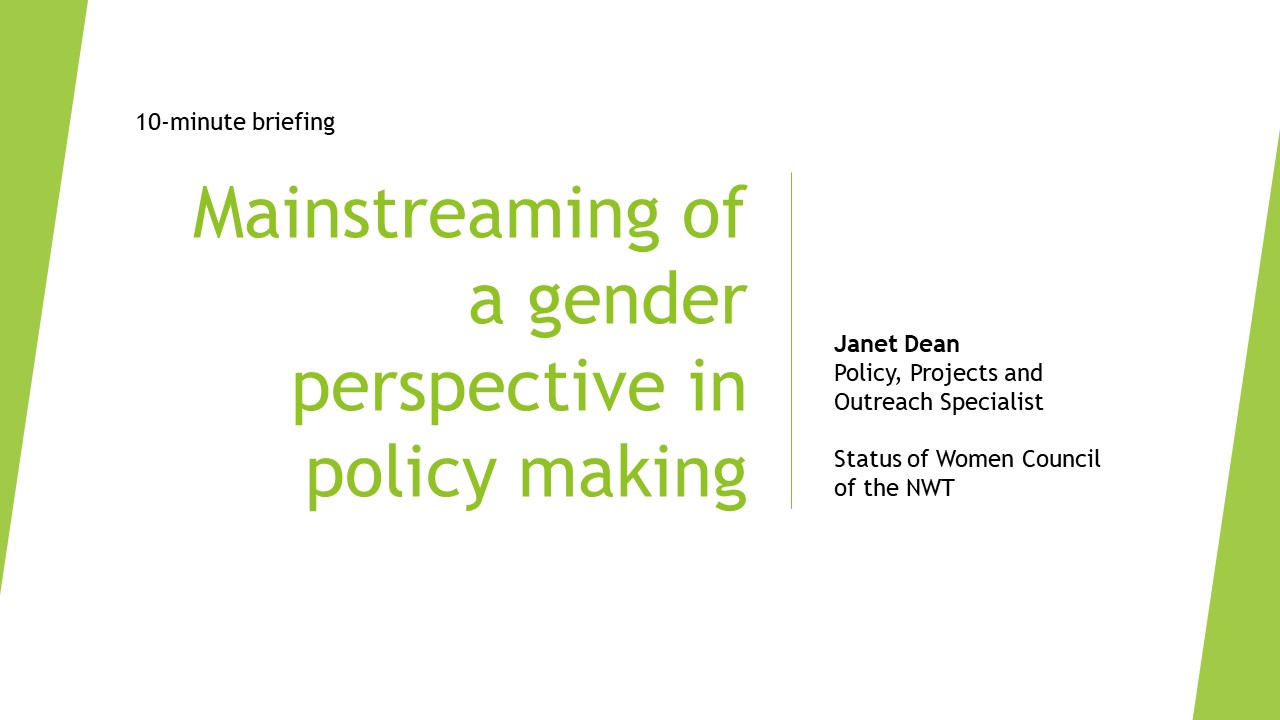

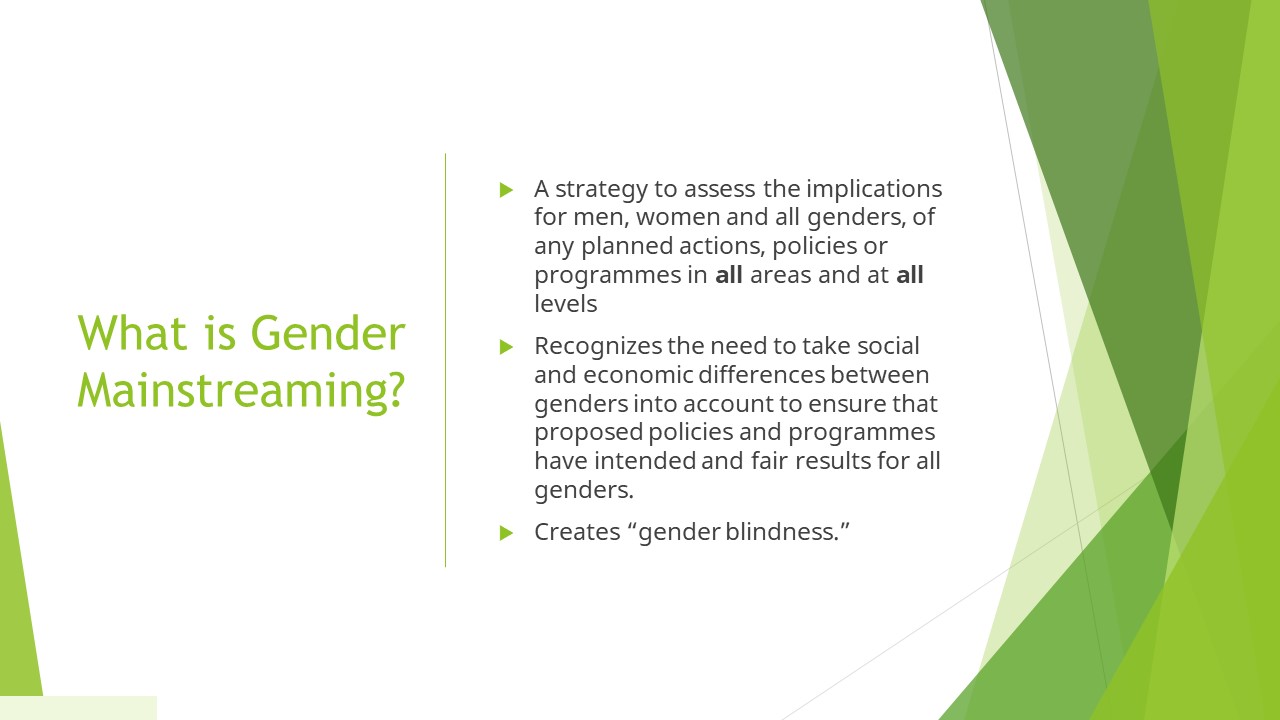

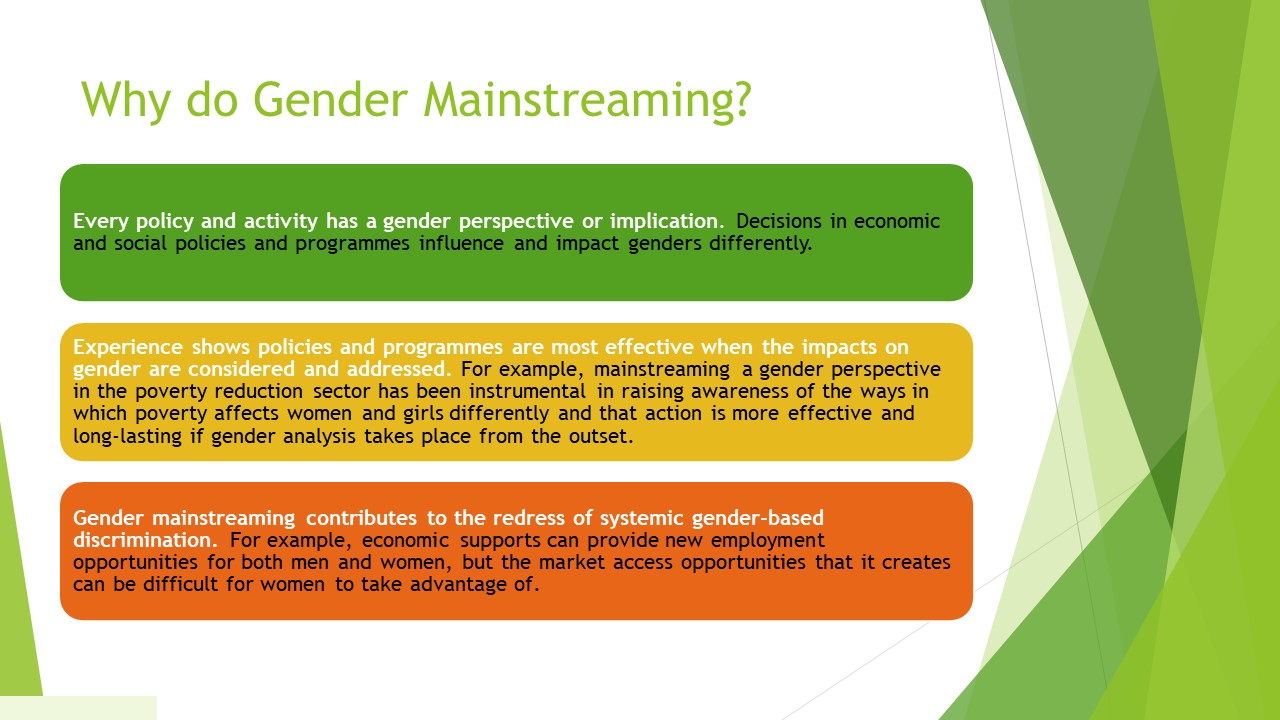

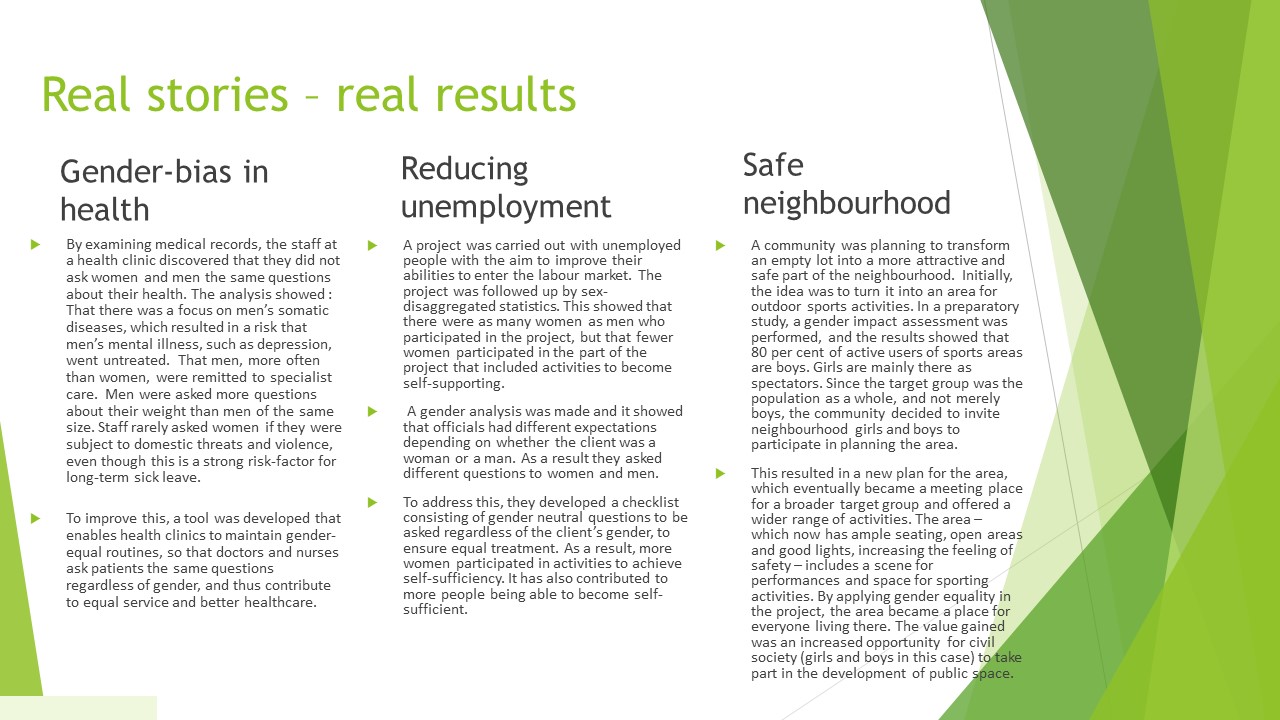

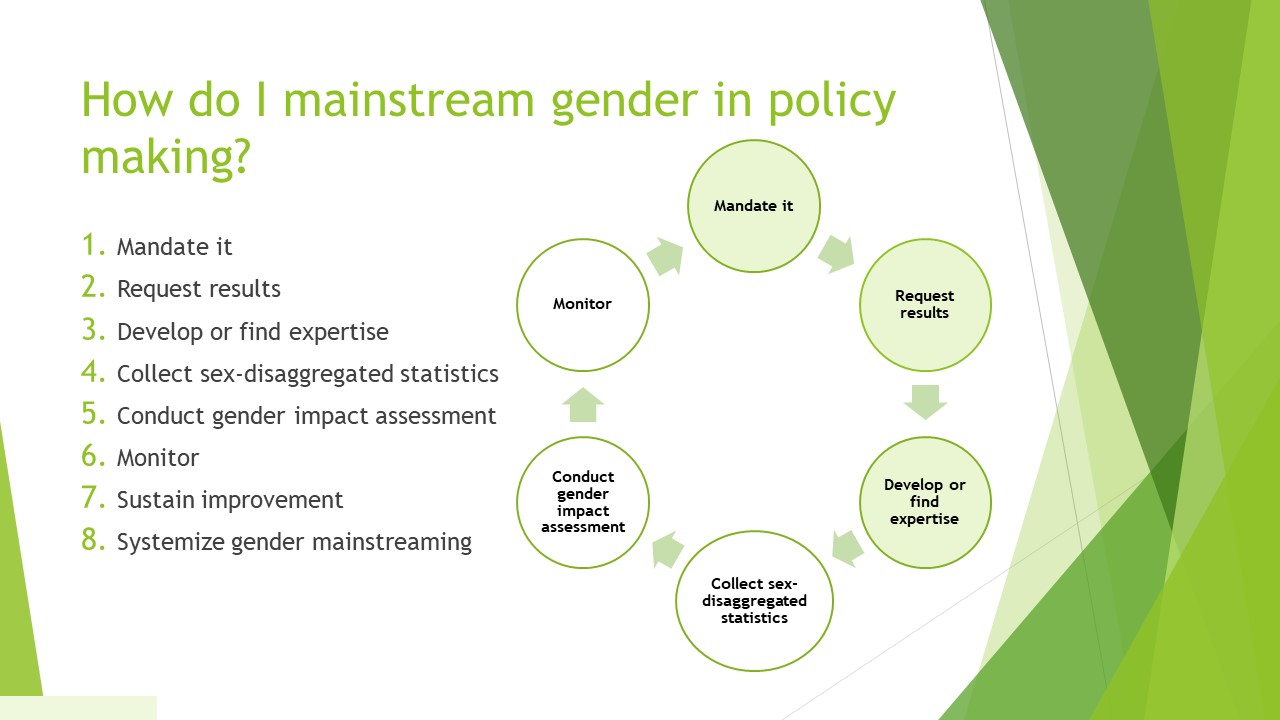

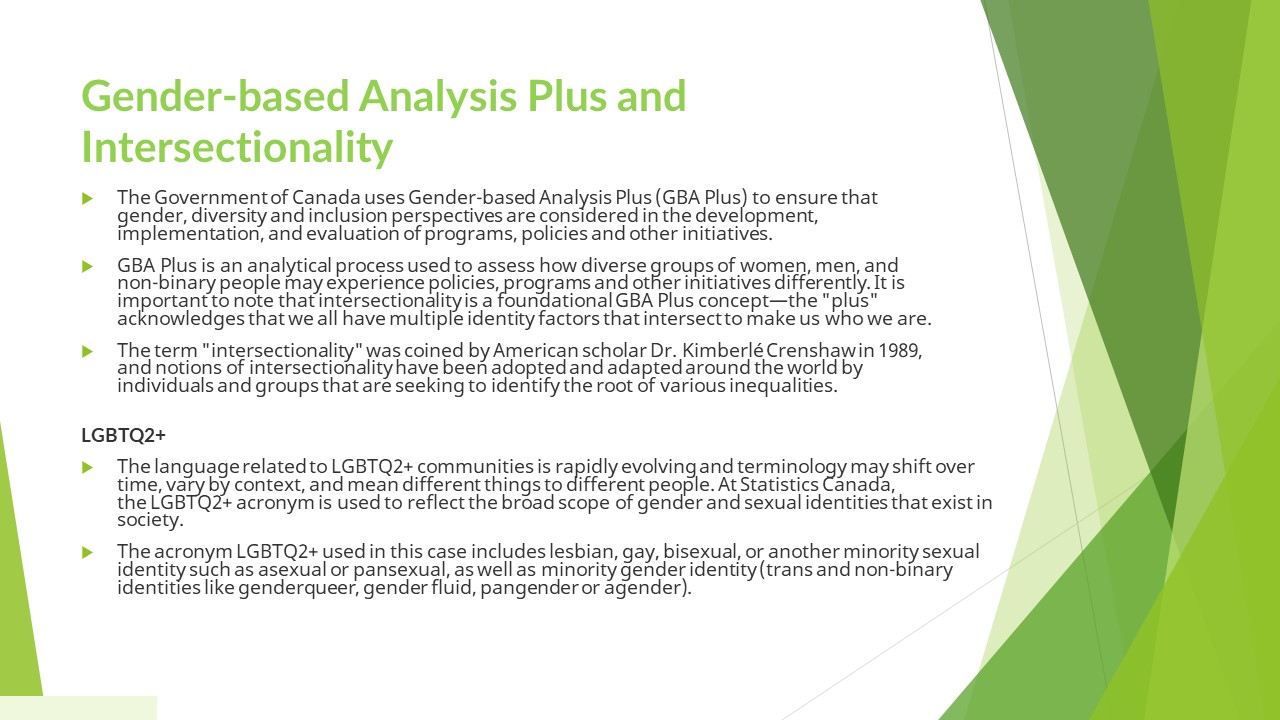

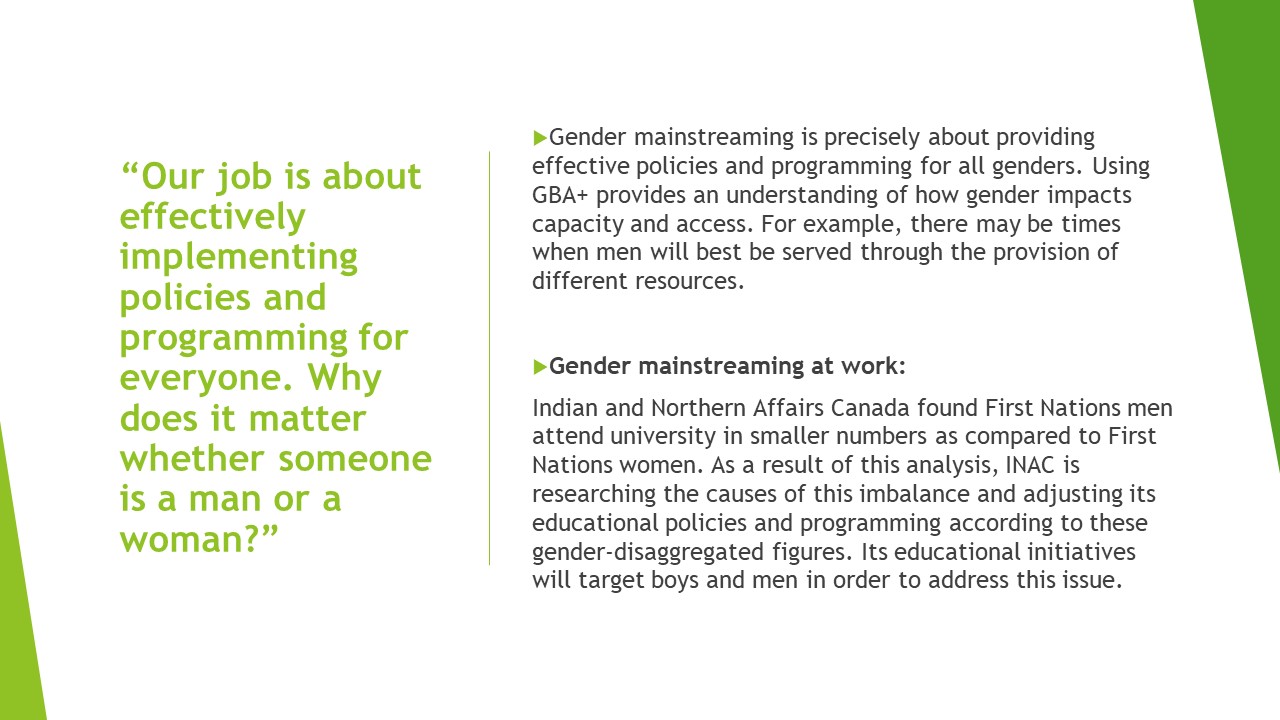

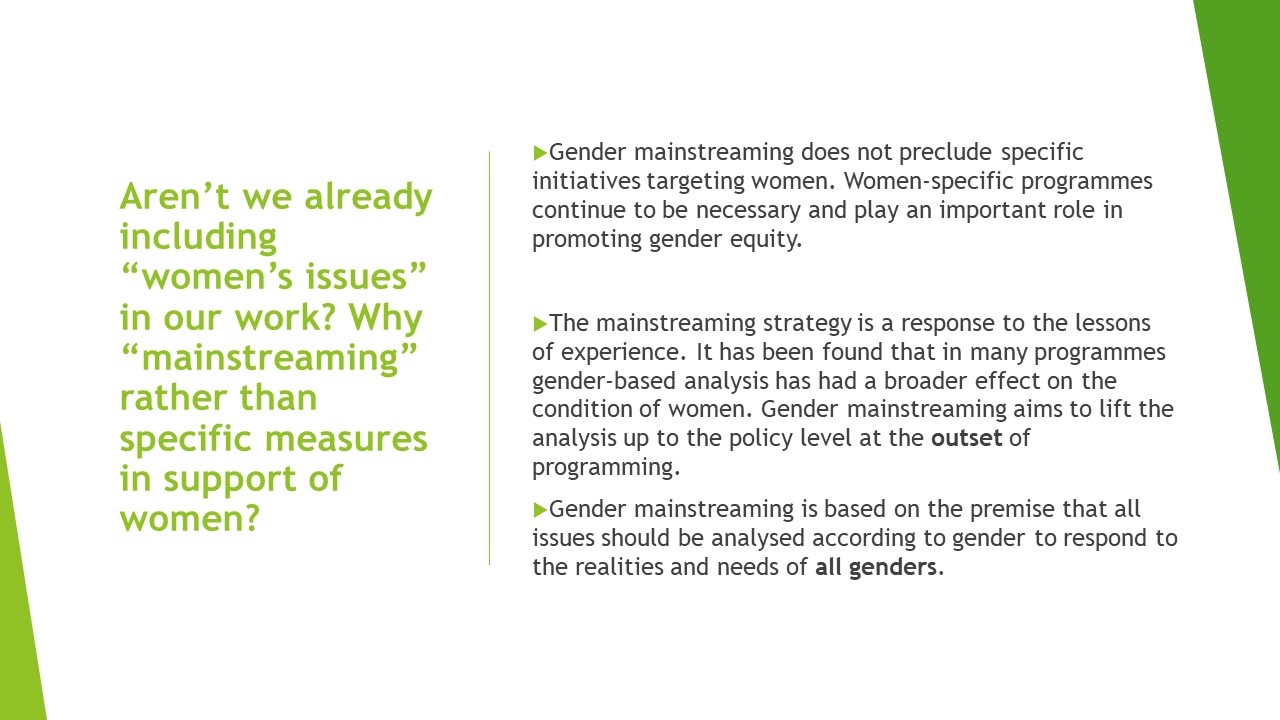

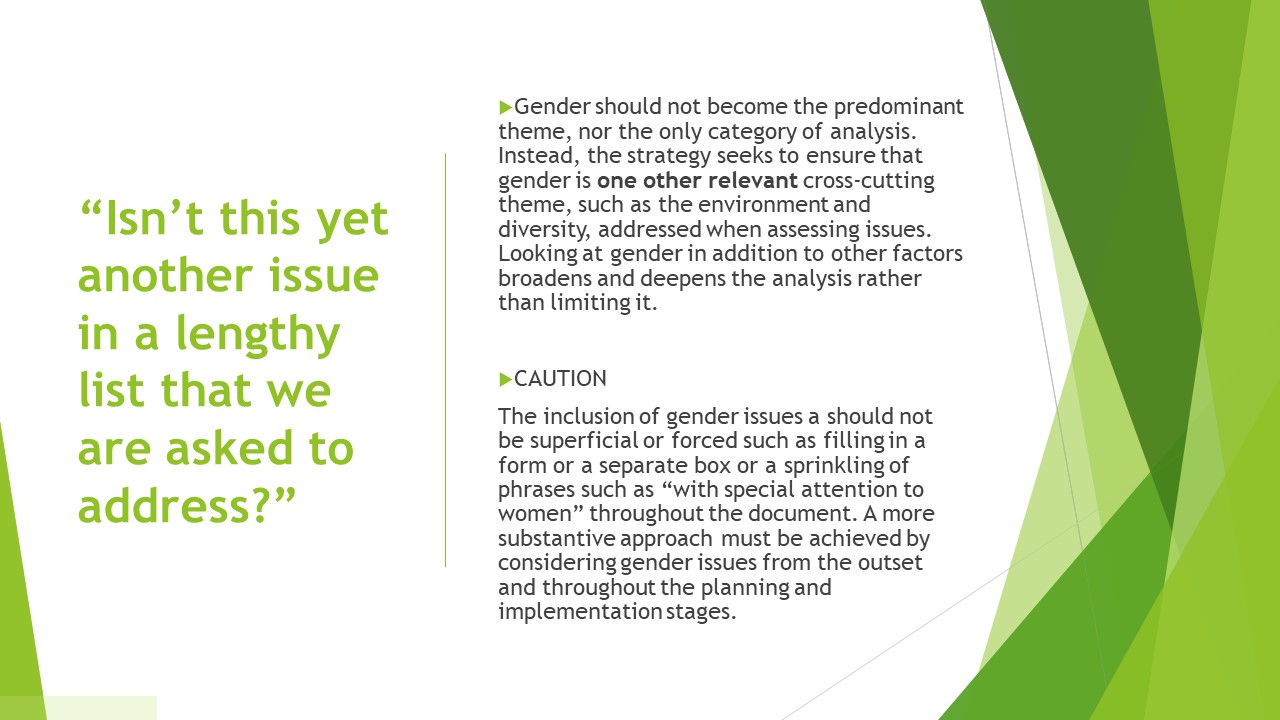

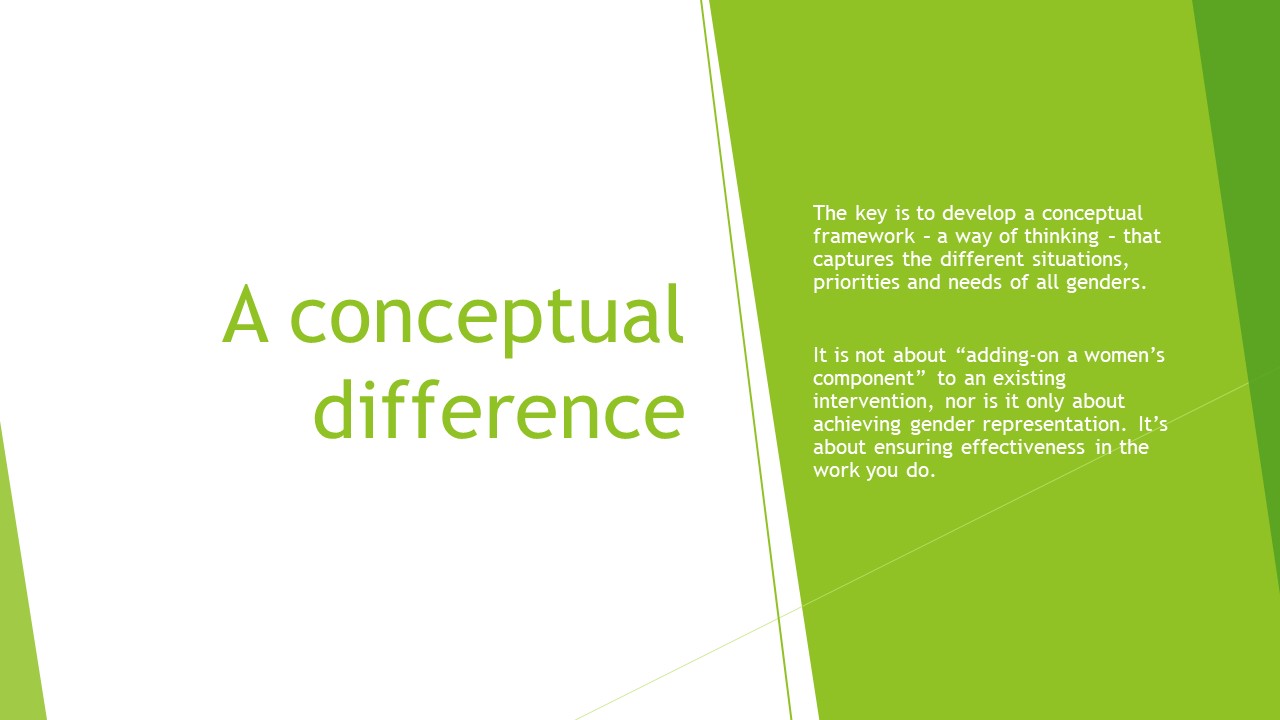

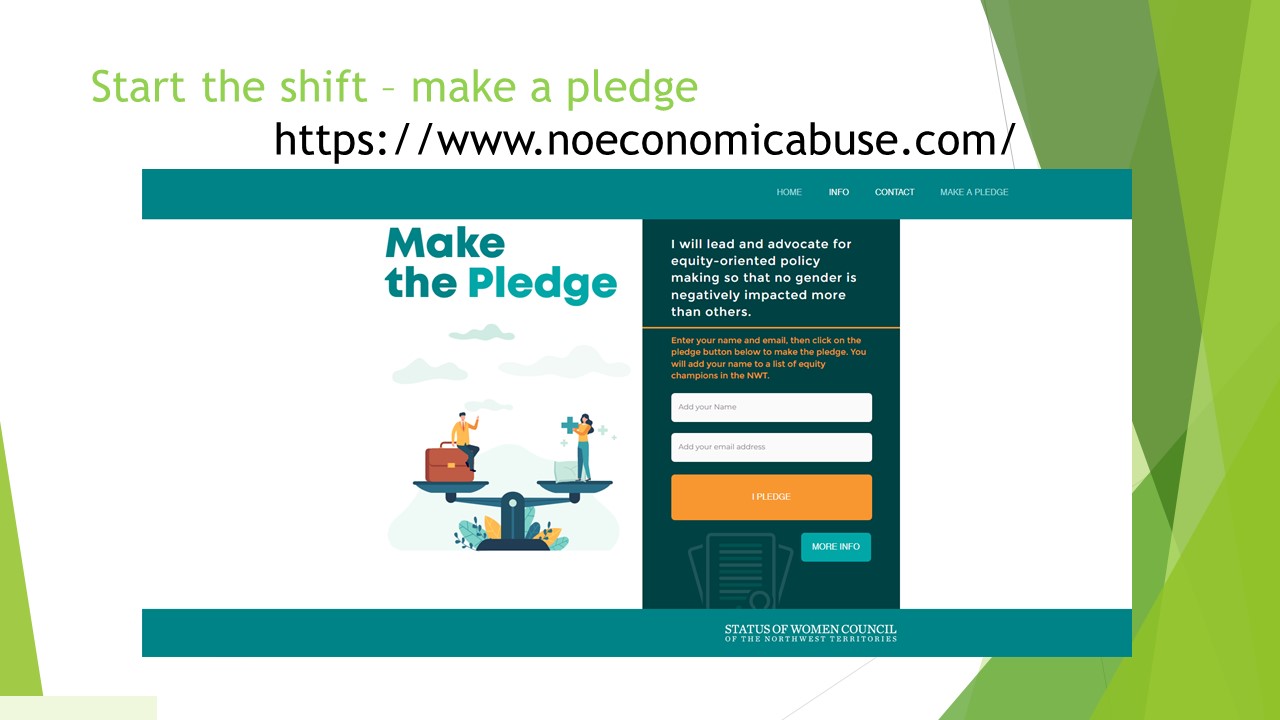

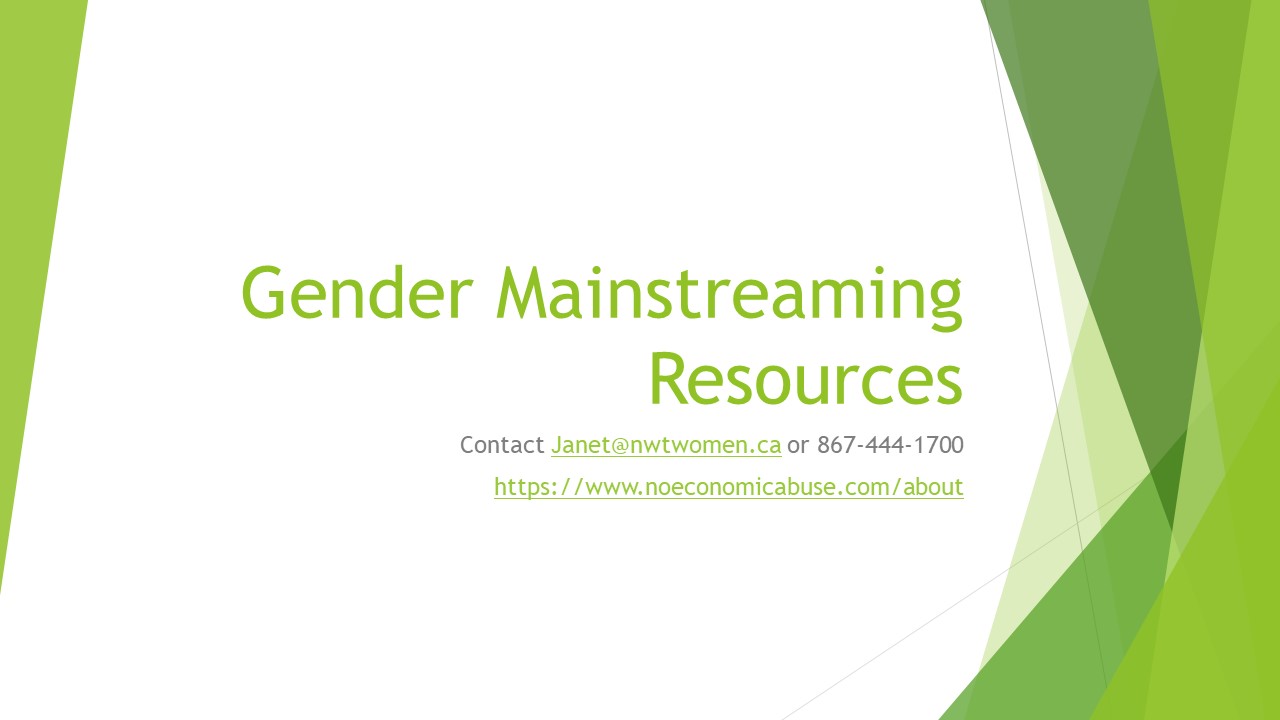
**
