# Supplementary material for: Nudging policymakers on gendered impacts of policy
Source: PLoS One. 2023 Oct 19;18(10):e0293036. doi: 10.1371/journal.pone.0293036 (PMC10586654; doi:10.1371/journal.pone.0293036)
Supplement: Attachment — Submitted filename: Response to Reviewers.docx [file pone.0293036.s002.docx]

*When submitting your revision, we need you to address these additional requirements.*

*1. Please ensure that your manuscript meets PLOS ONE's style requirements, including those for file naming. The PLOS ONE style templates can be found at*

*https://journals.plos.org/plosone/s/file?id=wjVg/PLOSOne_formatting_sample_main_body.pdf and*

[*https://journals.plos.org/plosone/s/file?id=ba62/PLOSOne_formatting_sample_title_authors_affiliations.pdf*](https://journals.plos.org/plosone/s/file?id=ba62/PLOSOne_formatting_sample_title_authors_affiliations.pdf)

The manuscript now meets PLOS ONE’S style requirements as outlined in the above documents.

*2. Please amend either the title on the online submission form (via Edit Submission) or the title in the manuscript so that they are identical.*

The title has been edited in the online submission form so that it is now identical to the manuscript.

*3. Please include captions for your Supporting Information files at the end of your manuscript, and update any in-text citations to match accordingly. Please see our Supporting Information guidelines for more information:* [*http://journals.plos.org/plosone/s/supporting-information*](http://journals.plos.org/plosone/s/supporting-information)*.*

Captions for supporting information files are now included at the end of the manuscript and in-text citations have also been updated to match.

**Reviewer #1 Comments**

*Thank you for the opportunity to review this manuscript. I have some concerns but overall I conclude this study is likely professionally executed and provides a well-evidenced challenge to some of the enthusiasm around what I characterise as ‘priming’ interventions to achieve social goals.*

Thank you for the positive remark and for your suggestions. We have addressed each of the comments below.

*My suggestions are to ensure that the result of the study fit with the wider literature and contribute to the broader debate.*

*First, I think this project would be better framed as a test of ‘priming policymakers’ rather than nudging policymakers. Why? Nudging is classically a libertarian paternalist notion. The key features of a nudge as introduced are:*

*1. The agency setting out the manipulation/intervention has some sort of authoritative position – as a governing body with a jurisdiction over the target or as an employer with some presumed interest the wellbeing of the target*

*2. The aim of the intervention is to improve the welfare of the target (as they would realistically or ideally see it themselves)*

*3. The targeted agent has the choice to do other than what the manipulation tries to get them to do (i.e. its not ‘hard paternalism’ or some sort of legal or contractual requirement)*

*See: Epstein RA (2018) The Dangerous Allure of Libertarian Paternalism. Review of Behavioral Economics 5(3–4): 389–416. DOI: 10.1561/105.00000087.*

*While 3. applies here (plenty of policymakers ignored the email and did not sign the pledge ultimately), points 1. and 2. do not apply. This is effectively a strategic communication or act of lobbying between actors that do not hold any specific authority over one another. Policymakers are constantly subject to multiple attempts to manipulate their behavior and priorities. The intervention is intended to contribute to the public good, not the private welfare of the policymaker. It is not a nudge: it is a prime.*

*The advantage of this framing is 1) it is more precise in terms of terminology; and 2) it highlights how much broader this result is as it challenges not only nudges but a whole category of manipulations of which some nudges are merely a sub-category.*

We thank the Reviewer for discussing the definition of nudge. While nudge often has the three characteristics of libertarian paternalism as pointed out by the Reviewer, we should point out that the nudge in our current study does not necessarily conform to this definition. Congiu and Moscati (2022) have outlined different types of nudges. One type is more traditional, called pro-self nudges, which are designed to benefit the nudged person and fit the characteristics #1 and #2. Another type of nudge, known as a pro-social nudge, is aimed at steering individuals towards behaviors that increase the common good but do not directly benefit the nudged person. Our nudge is a pro-social nudge. We designed the nudge with the Status of Women Council of the Northwest Territories (NWT) in Canada (an authoritative agency) to persuade policymakers in NWT to attend an online briefing on gendered impacts of policy. While this intervention does not directly benefit the policymakers, it is considered a pro-social nudge, because it can improve gender equity from government policies. We have clarified this definition of nudge in the revised introduction (p. 6). Our nudge is consistent with many examples of pro-social nudges in the past literature, including those that increase tax compliance (John & Blume, 2018), energy conservation (Allcott, 2011; Bernedo et al., 2014), and donations to charitable organizations (Pittarello et al., 2019). While these nudges do not directly benefit the nudged person, they are still considered to be nudges.

We do not think our interventions count as priming which is defined as using subconscious cues (physical, verbal or sensational) to drive choices (Friis et al., 2017; Wilson et al., 2016). Our interventions are fully explicit and elaborate and do not involve the use of subconscious cues.

References:

Allcott, H. (2011) Social norms and energy conservation. *Journal of Public Economics*, *95*(9–10), 1082–1095. DOI: [10.1016/j.jpubeco.2011.03.003](https://doi.org/10.1016/j.jpubeco.2011.03.003)

Bernedo, M., Ferraro, P. J., & Price, M. (2014) The persistent impacts of norm-based messaging and their implications for water conservation. *Journal of Consumer Policy*, *37*(3), 437–452. DOI: 10.1007/s10603-014-9266-0

Congiu, L., & Moscati, I. (2022). A review of nudges: Definition, justifications, and effectiveness. Journal of Economic Surveys, 36: 118-213. DOI: 10.1111/joes.12453

Friis, R., Skov, L. R., Olsen, A., Appleton, K. M., Saulais, L., Dinnella, C., ... & Perez-Cueto, F. J. (2017). Comparison of three nudge interventions (priming, default option, and perceived variety) to promote vegetable consumption in a self-service buffet setting. *PloS one, 12(5),* e0176028.

John, P., & Blume, T. (2018) How best to nudge taxpayers? The impact of message simplification and descriptive social norms on payment rates in a central London local authority. *Journal of Behavioral Public Administration*, *1*(1), 1–11. DOI: 10.30636/jbpa.11.10

Pittarello, A., Caserotti, M., & Rubaltelli, E. (2019) ‘Three is better than two’: Increasing donations with the attraction effect. *British Journal of Psychology*, *111*(4), 805–822. DOI: [10.1111/bjop.12428](https://psycnet.apa.org/doi/10.1111/bjop.12428)

Wilson, A. L., Buckley, E., Buckley, J. D., & Bogomolova, S. (2016). Nudging healthier food and beverage choices through salience and priming. Evidence from a systematic review. *Food Quality and Preference, 51,* 47-64.

*Second, this point could be further strengthened by more attention to the literature that led to the hypothesis generation for this experiment. How many of the studies discussed in the previous literature, I am thinking particularly in the Braddock & Dillard meta-analysis, involved pre-registered studies? If pre-registration has been uncommon in this area, then this might indicate various forms of publication or researcher bias have produced the previous results and this study (arguably superior in research design) is alerting behavioural scientists to be cautious. Pre-registration is a strength of this study that is worth emphasising against the general quality of the literature.*

Thank you for raising this point. Pre-registration does indeed seem to be uncommon in this area. We have expanded upon this point in the discussion section to more fully emphasize pre-registration as a strength of this particular study (p. 14).

*Smaller related points:*

*‘It is time to reorient the focus of nudge onto government itself — nudging policymakers directly to improve the way that policy is made’*

*Besides the use of nudge which I think is better re-characterised, this seems to involve unnecessary editorialising in a scientific study. The results of the study suggest that now might not be the time reorient narrative priming onto policymakers because here is some evidence it does not do all that much good. So reframing this as a question would show more alignment with the project.*

We have revised this sentence to “This raises the question about whether it is time to reorient…” (p. 3).

*Could the authors be more explicit when and how the policymakers consented to participate in the study? It would be good to know the full context about their knowledge they had about what was going on during the study.*

The last two paragraphs at the end of the email invitations (see Supporting Information) informed participants about the nature of the study and implied consent by participating in the study. The two paragraphs are:

“The Status of Women Council of the NWT has joined forces with a team from the University of British Columbia’s Behavioural Insights program to run a study exploring how policymakers and influencers consider important issues like gender equity. By participating in this study, you’ll play an active and visible role in improving the lives of all genders in the NWT. We are asking you to make a public declaration of your commitment to gender equity as part of this study.

If you have any questions, please reply to this email. You have been personally selected to receive this invitation. We ask that you please don’t share it with others at this time.”

We have made the consent process more explicit in the manuscript (p. 9).

*It could be worth caveating that one of the possible reasons for the treatment producing the opposite to the theorised effect is that it places the note about alternative times to see the webinar a lot further into the email than in the control group. I imagine the effectiveness of communications with policymakers depends a great deal on brevity and user-friendliness. The narratives might have disrupted these things. I do not see this as a direct problem for the results of the study as, if true, it merely indicates that narrative persuasion could be useful but not compared to more fundamental aspects of effective communication. Hence, it is another way of explaining the negative/null results.*

Thank you for raising this point. We have now included this possibility in the discussion section (p. 14-15).

**Reviewer #2 Comments**

*The paper provides interesting results of a policy evaluation process that is not often reported in policy papers. I commend the authors for reporting negative results. Below I detail what could be done to strengthen the paper.*

Thank you for the positive comments! We have addressed each point below.

*Comment 1: In line 58 in the introduction section page 3. The authors appear to suggest that gender equity has been overlooked by policymakers due to high information-processing demands. I believe this is not the only issue that may propagate inequities in gender. I propose that they include other reasons why gender equity is overlooked in addition to the issue of information.*

Thank you for this suggestion. We have added more discussion on why gender equity may be overlooked by policymakers in the introduction section: (p. 3-4)

“Other barriers include the lack of enforceable requirements on gender mainstreaming (defined as the process of assessing the impacts of any policies on women and men), often resulting in ‘lip service’ towards the principles of gender equality without the implementation of any practical changes [6]. Gender mainstreaming also challenges the status quo, resulting in limited implementation of gender equity strategies that challenge existing power structures[6,7]. Despite these system-level barriers, however, Payne [6] suggests that professional networks and authoritative experts can engage with policymakers in a number of formal and informal ways to influence policy development.”

*Comment 2: In terms of the pilot study, I wondered why the authors did not conduct a small qualitative study to explore the drivers behind the results depicted. The authors claim that failure to sign the pledge was because of a lack of sufficient information without any evidence. It would have been beneficial to have some qualitative component to explore the reasons and use that to design the second pilot.*

Due to resource constraints, we did not conduct a qualitative study to explore the drivers behind why policymakers did not sign the pledge. We think that the main driver was a lack of knowledge and hoped that our second pilot study where more information was provided through a briefing, would shed light on the issue. Unfortunately, no participant signed the pledge in the second pilot study, which led us to believe that the behavior of pledge signing may not be the most appropriate outcome measure.

*Comment 3: Based on the second pilot, I am wondering whether the choice of attendance rate as the primary behavioral measure should have been an intermediate outcome and retain the signing as the end result.*

In both pilot studies, no participant signed the pledge. This outcome made us think that while policymakers may want to know more about the issue, signing a public pledge may simply be too much of a commitment. Therefore, we decided to focus on attendance rate as our primary behavioral measure while keeping pledge signing as a secondary measure. While the attendance rate in the second pilot study was admittably low, we reasoned that this behavior is less consequential to policymakers than signing a public pledge, so we might see higher rates of attendance, but not signing. Unfortunately, due to time constraints, we were unable to do any further quantitative or qualitative testing before conducting the experiment.

*Comment 4: The authors reported that the responses were scripted, could the authors explain further, what this means? What if the participants asked a question that was outside the scripted responses? How was it handled?*

All questions from the participants were responded to with a scripted response indicating that they will be contacted after the briefing for a personal follow-up. If the question was outside the script, we would still respond to the participant in the follow-up. This has now been clarified in the manuscript (p. 10).

*Comment 5: were the participants who attended the policy briefing given any support on how to use the tool to implement the pledge in their policy work? This could be a gap that requires some attention.*

Yes we did. At the end of the briefing session, participants were emailed a link to a website where they could sign the pledge. This website also contained information on equity-oriented policymaking and gender mainstreaming. This information was designed to support policymakers on implementing the pledge in their own policy work. In addition, the website contained the contact information for the Status of Women Council of the Northwest Territories, so policymakers could contact the council if they had any questions (p. 10-11).

*Comment 6: One methodological issue I have with the authors is the scanty details provided on the qualitative component. I think they should detail the analysis process, the selection process of those whom they interviewed. There was also very limited information on the results with a tendency to present the summary quantitatively.*

The selection process for the qualitative follow-up involved contacting all participants by phone (n = 208) who did not respond to the initial email invitation. We were able to speak directly with 47 individuals and we asked them why they did not respond to the email invitations. From here, we simply tallied up the most common responses to try and get a sense of why participants did not respond to the emails. The manuscript has been revised to give more details of the analysis of the qualitative data (p. 12-13).

In addition to the qualitative phone calls, we report some anecdotal responses we received from participants throughout the course of the study, which were not systemtically collected. Wording in the manuscript has been revised to make it more clear that this was not part of the qualitative follow-ups (p. 13).

*Comment 7: In the discussion section, page 13 the authors report that the control group was more likely to attend the briefing than the treatment group. Although they provide information that was linked to context such as flooding etc, can the authors give an indication of the distribution of the 47 participants interviewed qualitatively from either control and intervention groups? This will provide a better sense of the reasons provided.*

One co-author has moved to a different organization and no longer had access to the raw qualitative data at the Status of Women Council. Thus, we can no longer perform any additional analyses on the qualitative data. This said, we have published our quantitative data which we still have.

*Comment 8: In page 14, the authors report on another qualitative conducted after the study, was this a different one from what was reported in this paper? If yes I suggest a better integration of the qualitative data throughout the paper with a detailed process of analysis*

We apologize for the confusion. The mention of qualitative data on page 15 is the same data we reported earlier in the paper. Wording has been revised to clarify this (p. 15).
